# Conservative Management of Suicide Left Ventricle After Surgical Aortic Valve Replacement

**DOI:** 10.7759/cureus.42890

**Published:** 2023-08-03

**Authors:** Hamza Chraibi, Lamyaa Bakamel, Rokya Fellat, Nesma Bendagha, Said Moughil

**Affiliations:** 1 Cardiology A Department, Ibn Sina Hospital, Mohammed V University, Rabat, MAR; 2 Cardiovascular Surgery B Department, Ibn Sina Hospital, Mohammed V University, Rabat, MAR

**Keywords:** left ventricular outflow tract obstruction, left ventricular hypertrophy, systolic anterior motion, cardiogenic shock, aortic valve replacement, suicide left ventricle

## Abstract

Suicide left ventricle (SLV) remains an underdiagnosed cause of haemodynamic compromise following surgical or transcatheter aortic valve replacement (AVR). Risk factors include female sex and a small left ventricular cavity with asymmetric septal hypertrophy. We present the case of a 63-year-old woman, with a medical history of diabetes mellitus, arterial hypertension and dyslipidaemia who was diagnosed with severe aortic stenosis with normal left ventricular ejection fraction and concentric hypertrophy. She underwent surgical AVR using a bioprosthetic valve, but a few hours after surgery, she developed sudden cardiogenic shock. An urgent transthoracic echocardiogram was performed showing marked systolic anterior motion of the mitral valve resulting in severe dynamic left ventricular outflow tract obstruction and intraventricular gradient. The diagnosis of SLV was made. The patient was managed conservatively with volume loading and oral beta-blockers and her haemodynamic state improved gradually. She was then discharged after favourable evolution. Medical management of SLV includes volume loading to expand the ventricular volume and beta-blockers for their negative inotrope effect. When medical therapy fails, surgical myectomy or alcohol septal ablation can be proposed to remove the obstacle. Some authors have proposed these procedures as prophylactic measures to prevent SLV in high-risk patients.

## Introduction

Suicide left ventricle (SLV) is a state of haemodynamic collapse provoked by dynamic left ventricular outflow tract obstruction (LVOTO) after a sudden afterload reduction. It most frequently happens after transcatheter aortic valve replacement (TAVR) [1] but reported cases after surgical aortic valve replacement (SAVR) are few and far between [2-4]. In many cases, mitral systolic anterior motion (SAM) contributes to the LVOTO, which may result in elevated gradients. A redo surgery, such as an edge-to-edge repair, is often indicated to correct structural mitral valvular issues, but these procedures are correlated with a higher morbidity and mortality risk [3,5]. 

In this paper, we describe a case of SLV associated with SAM following SAVR which was successfully managed with no need of reoperating the patient. We discuss the pathophysiology of SLV as well as its management options.

## Case presentation

**Figure 1:**
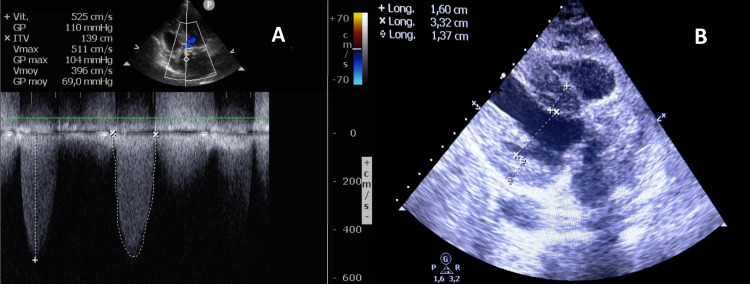
Preoperative transthoracic echocardiogram showing severe aortic stenosis A: Continuous-wave Doppler signal showing a transaortic mean gradient of 69 mmHg and a maximum velocity of 5.1 m/s. B: Asymmetrical left ventricular septal hypertrophy, measured at 16 mm.

A 63-year-old woman, with a medical history of diabetes mellitus, arterial hypertension and dyslipidaemia, complained of angina and exertion dyspnoea beginning three years before. Auscultation found a systolic murmur in the aortic area with an absent S2. Her electrocardiogram showed sinus rhythm with left ventricular (LV) and atrial hypertrophy. Transthoracic echocardiogram (TTE) revealed severe aortic stenosis with a mean gradient of 69 mmHg and an aortic valve area of 6 mm² (Figure 1A). LV ejection fraction was normal and there was concentric LV hypertrophy with a septal thickness of 16 mm (Figure 1B).

**Figure 2:**
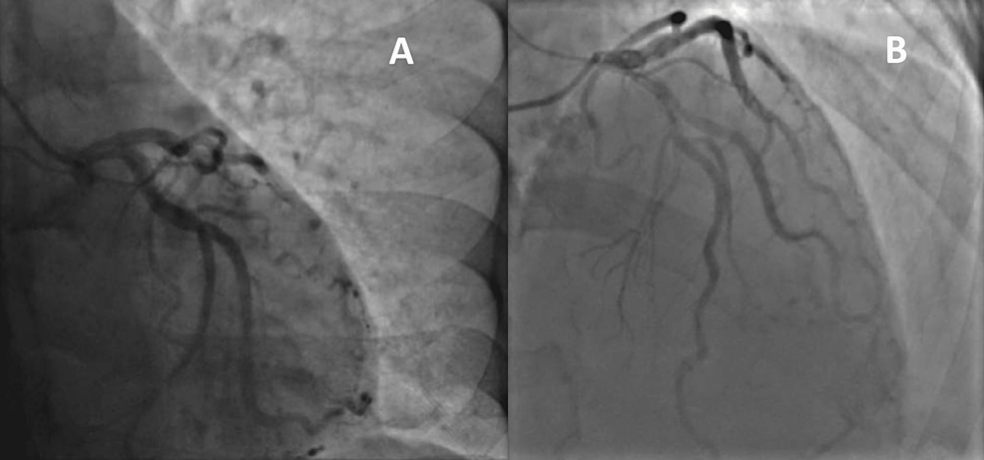
Coronary angiogram showing normal coronary arteries A: Right oblique anterior view. B: Left oblique anterior view.

The heart team’s decision was immediate SAVR. The preoperative coronary angiogram was normal (Figure 2).

**Figure 3:**
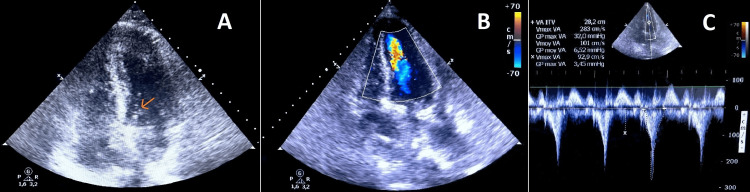
Immediate postoperative transthoracic echocardiogram A: Mitral valve systolic anterior motion (arrow). B: Intraventricular aliasing. C: Dagger-shaped spectral Doppler with a peak gradient of 32 mmHg.

The surgery was completed uneventfully with the implantation of a 21 mm bioprosthetic valve (Edwards Lifesciences). When transferred to the intensive care unit (ICU), the patient was initially stable. A few hours later, she developed sudden cardiogenic shock, marked by severe refractory hypotension despite normetanephrine perfusion. An urgent TTE was performed showing marked SAM of the mitral valve (Figure 3A), resulting in severe dynamic LVOTO. Intraventricular aliasing was present (Figure 3B) and the spectral Doppler was dagger-shaped with a peak gradient of 32 mmHg and a maximum velocity of 2.8 m/s (Figure 3C), confirming the diagnosis of SLV.

**Figure 4:**
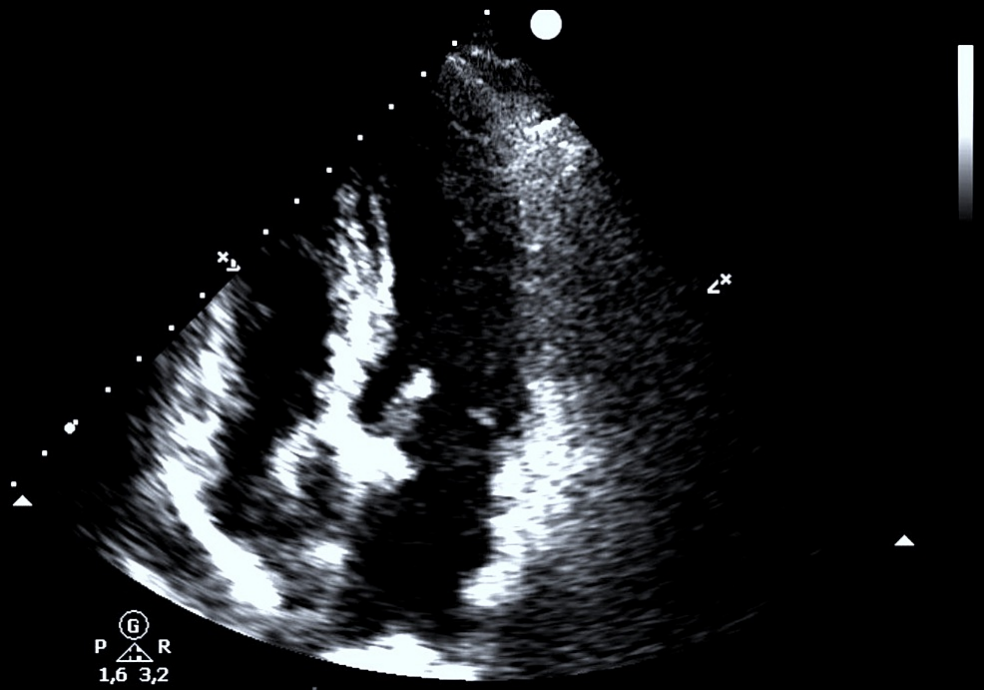
Postoperative TTE showing the bioprosthetic aortic valve with the resolution of mitral SAM TTE: transthoracic echocardiogram; SAM: systolic anterior motion.

The patient was managed conservatively with volume loading and oral beta-blockers and her haemodynamic state improved gradually. She was transferred from the ICU three days later and a control TTE found a good function of the bioprosthetic valve with no SAM (Figure 4).

## Discussion

Aortic stenosis is a slowly progressive disease, giving the heart ample time to adapt to this chronic increase in afterload. LV hypertrophy and impaired relaxation are the hallmarks of this adaptation phenomenon [6]. After a sudden afterload reduction (i.e. TAVR or SAVR), a patient’s course may, therefore, be marked by a labile haemodynamic state resulting in high dynamic intraventricular gradients (DIGs). The prevalence of high DIG ranges from 14% to 25% following SAVR [7,8]. In the most severe cases such as our patient’s, SAM and LVOTO result in low cardiac output and haemodynamic compromise. SLV, therefore, remains an underdiagnosed cause of post-SAVR cardiogenic shock and should be systematically ruled out [9].

SLV most commonly happens after AVR and has even been reported after mitral valve replacement [10]. The main predisposing factor to post-SAVR LVOTO is unfavourable LV anatomy. Typically, the LV is hyperdynamic, with a small cavity and a narrow outflow tract, and hypertrophied. Patients with asymmetrical septal hypertrophy, as was the case in our report, are especially at risk. Other predisposing factors include but are not limited to female sex and postoperative hypovolemia [5,7-10].

After the diagnosis of SLV is made, the immediate goal is to reduce LVOTO. The cornerstones of medical therapy are volume loading to expand the ventricular cavity and beta-blockers for their negative inotrope effect. Vasoconstrictors such as phenylephrine could be beneficial as they increase systemic vascular resistance. Diuretics should be discontinued. When medical therapy fails, surgical myectomy or alcohol septal ablation can be proposed to remove the obstacle. Some authors have proposed these procedures as prophylactic measures to prevent SLV in high-risk patients [11,12].

## Conclusions

This case report highlights a very rare cause of haemodynamic collapse following SAVR. The diagnosis of SLV is relatively quick and simple, requiring a single bedside TTE. Its medical management, based on volume loading and beta-blockers, is completely different from other aetiologies of cardiogenic shock. Cardiologists, cardiothoracic surgeons and intensivists should be well aware of this complication and its risk factors.
